# Synthesis and Biological Evaluation of Pyrimidine-oxazolidin-2-arylimino Hybrid Molecules as Antibacterial Agents

**DOI:** 10.3390/molecules23071754

**Published:** 2018-07-17

**Authors:** Roberto Romeo, Maria A. Chiacchio, Agata Campisi, Giulia Monciino, Lucia Veltri, Daniela Iannazzo, Gianluigi Broggini, Salvatore V. Giofrè

**Affiliations:** 1 Dipartimento di Scienze Chimiche, Biologiche, Farmaceutiche ed Ambientali, University of Messina, Via S.S. Annunziata, 98168 Messina, Italy; sgiofre@unime.it; 2 Dipartimento di Scienze del Farmaco, Università di Catania, Viale A. Doria, 95100 Catania, Italy; ma.chiacchio@unime.it (M.A.C.); agcampisi@gmail.com (A.C.); gmonciino@unict.it (G.M.); 3 Dipartimento di Chimica e Tecnologie Chimiche, Università della Calabria, Via P. Bucci, 12/C, 87036 Arcavacata di Rende, Italy; lucia.veltri@unical.it; 4 Dipartimento di Ingegneria, Università di Messina, Contrada Di Dio, 98166 Messina, Italy; diannazzo@unime.it; 5 Dipartimento di Scienza e Alta Tecnologia, Università dell’Insubria, via Valleggio 11, 22100 Como, Italy; gianluigi.broggini@uninsubria.it

**Keywords:** intramolecular alkoxyhalogenation, Suzuki coupling, antibacterial compounds, pyrimidine-oxazolidinone hybrids, molecular docking

## Abstract

Pyrimidine-1,3-oxazolidin-2-arylimino hybrids have been synthesized as a new class of antibacterial agents. The synthetic approach exploits a Cu(II)-catalyzed intramolecular halkoxyhalogenation of alkynyl ureas, followed by a Suzuki coupling reaction with 2,4-dimethoxypyrimidin-5-boronic acid. Biological screenings revealed that most of the compounds showed moderate to good activity against two Gram-positive (*B. subtilis*, *S. aureus*) and three Gram-negative (*P. aeruginosa*, *S. typhi*, *K. pneumonia*) pathogenic strains. A molecular docking study, performed in the crystal structure of 50S ribosomal unit of *Haloarcula marismortui,* indicated that pyrimidine-oxazolidin-2-arylimino hybrids **8c** and **8h** exhibited a high binding affinity (−9.65 and −10.74 kcal/mol), which was in agreement with their good antibacterial activity. The obtained results suggest that the combination of pyrimidine and oxazolidone moieties can be considered as a valid basis to develop new further modifications towards more efficacious antibacterial compounds.

## 1. Introduction

**Figure 1:**
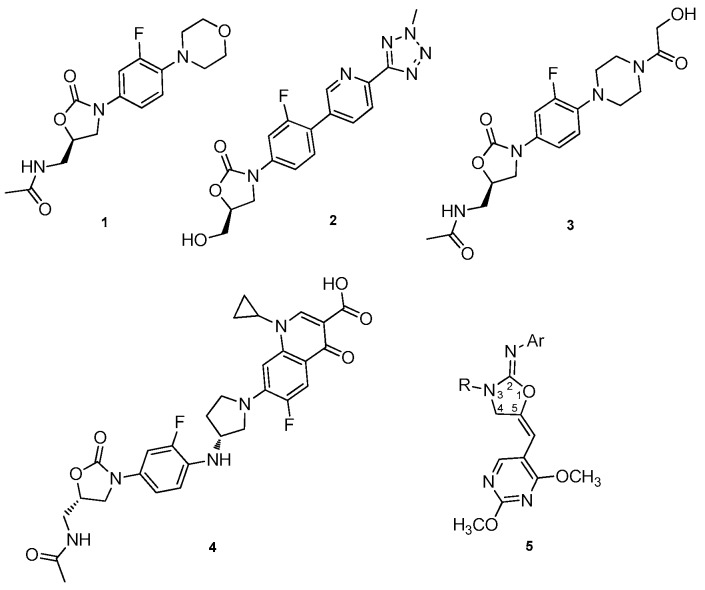
Oxazolidinone-based antibacterial agents.

The oxazolidinone unit is the important core structure of a class of synthetic antibacterial agents that possess activity against a variety of Gram-positive pathogenic bacterial strains and are highly potent against multidrug resistant bacteria [1,2,3,4,5,6,7,8]. Linezolid **1**, the first oxazolidinone antibiotic clinically approved [9], and the strictly correlated Eperezolid **2** [5,10] and Tedizolid **3** (Figure 1) target the bacterial ribosome by inhibiting protein synthesis and preventing the initiation of mRNA translation [7,10].

Despite showing considerable clinical promise, these compounds suffer some limitations that prevent the general use of the drug. Linezolid is associated with undesirable side effects, such as thrombocytopenia, myelosuppression, neuropathies, and bone marrow toxicity [11,12,13,14,15] due to the inhibition of mammalian mitochondrial protein synthesis. Moreover, because of the inhibitory effects on monoaminoxidase [14,15], treatment with Linezolid or Tedizolid may lead to unfavorable interactions with serotonergic and adrenergic agents and, then, to severe hypertensive crisis [16,17].

Linezolid and many other oxazolidinones do not show good activity towards Gram-negative bacteria. Furthermore, the development of antimicrobial resistance represents a serious health problem and contributes strongly to the urgent need for the discovery of new effective agents in this area.

Accordingly, continuous efforts have been addressed to develop a new generation of oxazolidinone-based antimicrobials with the aim of lowering the toxicity potential, improving the potency and broadening the antibacterial spectrum of this class of compounds by extending the activity to Gram-negative bacteria and mycobacteria.

Besides modifying the substitution pattern on the core structure, another fruitful type of approach is the hybridization of oxazolidinones with other classes of antimicrobial compounds: the combination of two classes of drugs could yield new antibacterial agents with desired properties. Interestingly, different oxazolidinone derivatives when linked with active heterocyclic pharmacophores play an important role against Gram-positive infections: [18] for example, an oxazolidinone-quinolone hybrid **4** (Figure 1) displayed very good antimicrobial properties when two pharmacophoric groups are incorporated together [19].

Heterocyclic units play a fundamental role in the design and development of new classes of compounds exploitable for medicinal applications. In particular, pyrimidine and its derivatives occupy a key place in medicinal chemistry due to their diverse biological activities and their presence in several biologically active natural products. Synthetic pyrimidine-based scaffolds have shown to be endowed with a wide spectrum of chemotherapeutic activities such as anti-inflammatory [20], antiparasitic [21], anti-allergic [22], and antitumor activities [23]. In addition, pyrimidines are potential inhibitors of dihydrofolate reductase (DHFR), a promising drug target for the development of anti-infective agents [24].

On the basis of these considerations, we have envisaged that the synthesis of new pyrimidine-oxazolidinone hybrids could gain advantage from the insertion of dual pharmacophores in a single molecular framework.

According to this hypothesis, we have designed a new scaffold of pyrimidine- oxazolidone analogues, wherein the active oxazolidinone unit is linked at its 5-position to the 5-position of the pyrimidine ring, through a methyne bridge: the conversion of the keto group at position 2 of the oxazolidinone ring into an *N*-arylimino unit has been suggested by docking data in order to improve the interaction with the active site of the enzyme. Furthermore, many researchers have studied the activity of Schiff bases as potent antibacterial agents with FabH inhibiting activity [25,26]. Thiazole, morpholine and more recently linezolid-like Schiff bases have been reported as inhibitors of *Pseudomonas aeruginosa* [27,28].

In this communication, we report the synthesis of hitherto unknown title compounds **5** (Figure 1), starting from easily accessible alkynyl ureas, by exploiting an intramolecular oxidative Cu-catalyzed alkoxyhalogenation process [29], followed by a Suzuki reaction with 2,4-dimethoxypirimidin-5-boronic acid.

All the synthesized compounds have been evaluated in vitro for their antibacterial activity against two Gram-positive (*Bacillus subtilis* MTCC121, *Staphylococcus aureus* (MTCC96) and three Gram negative (*Pseudomonas aeruginosa* MTCC741, *Salmonella typhi* MTCC537, *Klebsiella pneumonia* MTCC3384) bacterial strains. The potential antifungal activity has been also tested against two fungal species, *Candida albicans* MTCC 3017, and *Candida tropicalis* MTCC184. Docking data support the biological results: molecular modeling experiments have been performed based on the *Haloarcula marismortui* 50S ribosomal subunit.

## 2. Results and Discussion

### 2.1. Chemistry

**Scheme 1:**
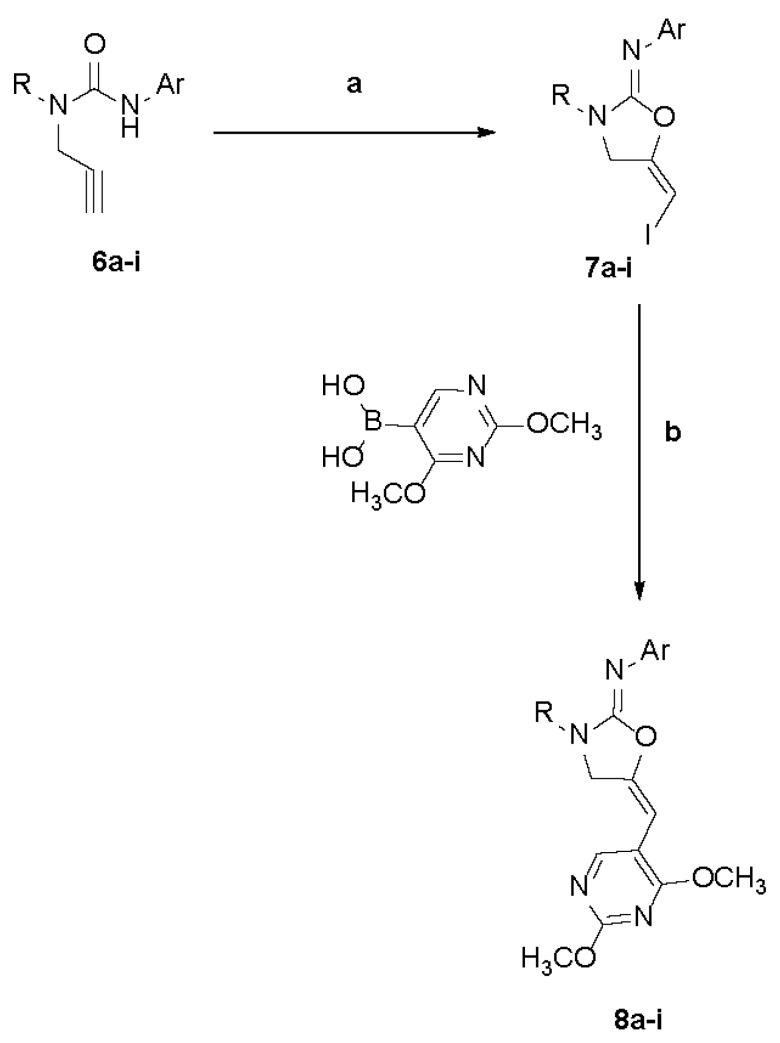
Reagents and conditions: (a) CuI_2_, *N*-iodosuccinimide (NIS), MeCN, 7h, 70 °C, rt., overnight; (b) Na_3_PO_4_, Pd(dppf)Cl_2_.CH_2_Cl_2_, THF, 60 °C, overnight.

**Figure 2:**
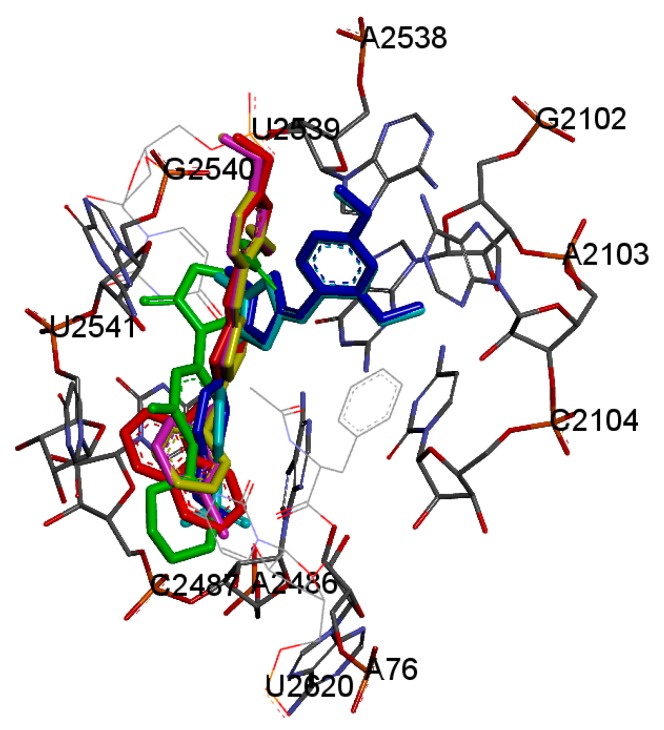
Binding mode of the **8a**–**e** series. Linezolid (green), **8a** (yellow), **8b** (red), **8c** (cyano), **8d** (magenta), **8e** (blue).

5-[(2,4-Dimethoxypyrimidin-5-yl)methylene]oxazolidin-2-arylimines **8** were prepared through the route illustrated in Figure 2. The synthetic approach proceeds through two steps. Thus, the alkynylureas **6a**–**i**, prepared by reaction of the alkynyl amine with the suitable isocyanate [29], were reacted with a catalytic amount of CuI_2_ in the presence of a stoichiometric amount of *N*-iodosuccinimide (NIS) to give in good yields the corresponding iodooxazolidine derivatives **7a**–**i** (Scheme 1, Table 1) [29].

As reported [29], the mechanism of the alkoxyhalogenation reaction is based on the formation of a π-complex between the triple bond and CuI_2_ which promotes the exo-dig cyclization by nucleophilic attack on the activated triple bond. The subsequent deprotonation and the reaction with NIS provides the final product **7** with regeneration of the copper catalysts.

The obtained oxazolidine derivatives **7** were then converted into the title compounds **8** by Suzuki coupling reaction performed with 2,4-dimethoxypyrimidin-5-boronic acid in the presence of Pd(dppf)Cl_2_.CH_2_Cl_2_, K_3_PO_4_ in THF at 60 °C for 12 h (Scheme 1; Table 2).

The structure of the obtained compounds was assigned on the basis of spectroscopic data. In particular, the ^1^H NMR spectra of **8h**, chosen as model compound, shows the pyrimidine methoxy groups as singlet at 3.98 and 3.97 ppm, and the methylene protons of the oxazolidine ring as a doublet at 4.22 ppm. The vinylic proton gives rise to a triplet at 6.20 ppm, while pyrimidine ring proton resonates as a singlet at 7.88 ppm.

On the other hand, in the ^13^C NMR spectra, the resonance of the methylene group of the five-membered ring, in the range 47.7–48.0 ppm, is consistent with the 4,5-dihydro-oxazole ring, while the benzylic methylene group is at 47.6. The vinylic carbon and the two methoxyl groups resonate at 95.6, 54.8 and 54.1 ppm respectively.

The *E* configuration of the exocyclic double bond has been assessed by Nuclear Overhause Effect (NOE) experiments. Thus, the irradiation of methyne proton at 6.20 ppm induces a positive NOE Effect on the pyrimidine ring proton resonance at 7.88 ppm.

### 2.2. Antimicrobial Evaluation

All the synthesized compounds **8a**–**i** were screened for their in vitro antibacterial activity against two Gram-positive [*Bacillus subtilis* (Bs) MTCC121, *Staphylococcus aureus* (Sa) MTCC96], and three Gram-negative [*Pseudomonas aeruginosa* (Pa) MTCC741, *Salmonella typhi* (St) MTCC537, *Klebsiella pneumonia* (Kb) MTCC3384] bacterial strains.

Antifungal activity was screened against two fungal species [*Candida albicans* (Ca) MTCC 3017, *Candida tropicalis* (Ct) MTCC184].

The minimal inhibitory concentrations (MICs) of all active compounds **8a**–**i** were determined by micro broth dilution method using 96 well plates, according to the method described [30]. Linezolid, ciprofloxacin and fluconazole have been used as standards; DMSO has been employed as solvent control. The results of antimicrobial screening are summarized in Table 3.

Cell viability tests have been performed in order to evaluate the antibacterial toxicity of the most active compounds.

### 2.3. Biological Results

According to our initial hypothesis, the oxazolidinone unit linked to the pyrimidine pharmacophore appears to play an important role against the bacterial infections. The investigation of antimicrobial screening data (Table 3) revealed that most of the tested compounds **8a**–**i** showed moderate to good inhibitory activity against Gram-positive bacteria *Bacillus subtilis* and *Staphylococcus aureus*.

The inhibition activity has been found to be dependant upon the various substituents present on the aromatic ring (Ar). In particular, two compounds, **8c** and **8h**, showed the highest potency, with MIC in the range 2.8–4.8 μg/mL as compared to Linezolid (3.0) and ciprofloxacin (3.5). Compounds **8a**, **8b**, **8f** and **8g** exhibited a lower activity, with MIC in the range 9.5–45 μg/mL.

A similar trend has been observed in the case of inhibiting Gram negative bacteria: compounds **8c** and **8h** were also found to be the most potent derivatives with MIC values in the range 9.5–21 μg/mL. In all the determinations, tests were performed in triplicate and the results were taken as a mean of three determinations.

The comparison of inhibition among the different compounds suggests that the presence of a *p*-NO_2_ group on the arylimino moiety (compounds **8c** and **8h**) leads to maximum inhibition against the examined bacterial strains. On the contrary, a strong electron-donating substituent as OMe, at the same position, strongly reduces the biological activity.

The obtained results clearly suggest that compounds **8** may represent a new class of antibacterial agents in future. Further studies may be extended for their applications as the active pharmaceutical agents.

The result of antifungal study (Table 3) for all the synthesized derivatives revealed that the compounds had poor activity against *Candida tropicalis* (Ct), whereas compounds **8c** and **8h** contributed moderate antifungal activity against *Candida albicans* (Ca). All other compounds had weak or absent antifungal potency.

The cytotoxicity of the most bioactive compounds was evaluated in vitro against human dermal fibroblast (HDF) cell line using the colorimetric cell proliferation MTT (3-(4,5-Dimethylthiazol-2-yl)-2,5-Diphenyltetrazolium Bromide) assay [31]: as shown in Table 4, tested compounds exhibited relative low toxicity at high concentrations, suggesting a great potential for their use as antimicrobial agents (Table 4).

Known numbers of cells (1.0 × 10^−4^) were incubated for 24 h in a 5% CO_2_ incubator at 37 °C with different concentrations of test compounds. After 24 h of drug incubation, MTT solution was added, supernatant was discarded, 100 mL DMSO were added in each well and absorbance was recorded at 540 nm by ELISA reader.

### 2.4. Molecular Docking Study

Oxazolidinones exhibit their antibacterial features by inhibiting the protein biosynthesis by binding to sites on the bacterial ribosomes, thus preventing the formation of a functional 70S initiation complex [32,33]. Linezolid binds to the A-site of the 50S subunit [11] thus preventing the binding of the aminoacyl-tRNA.

Accordingly, to understand the possible binding mode and the structure-activity relationship of new pyrimidine-oxazolidin-2-arylimino hybrids **8a**–**i**, molecular docking study of selected compounds was performed in the crystal structure of 50S ribosomal unit of *Haloarcula marismortui* (PDB code 3CPW) [10] using the AutoDock package.

Most ribosomal structures derive from either halophilic archaebacteria (*H. marismortui*) or extremophilic bacteria (*Thermus thermophilus* and *Deinococcus radiodurans*). In particular, the resolution of the complex of *H. marismortui* 50S subunit with linezolid has provided important insight of the interaction mechanism [10]: thus, we have chosen the crystal structure of the canonical oxazolidinone, as linezolid, bound to *H. marismortui* to define the binding mode of our compounds as plausible bacterial inhibitors. The sequence of the 50S ribosomal subunit of *Haloarcula marismortui* showed good similarity (78%) with that of other strains, such as *D. radiodurans* (D50S) (78%) and Escherichia coli (77%) [34] based on sequence alignment using the BLASTN 2.2.29+ software [35]. Furthermore, sequence alignments showed that the regions of the 50S structures discussed in this study are highly conserved, so the structural rationales proposed would be expected to hold for *S. aureus* and other species we have reported in this paper [36].

The docking protocol was validated by redocking method of cocrystallized structure in the binding site to determine the lowest RMSD (root mean square deviation) relative to the crystallographic pose. Linezolid, was successfully redocked with a RMSD of 0.76 Å.

**Figure 3:**
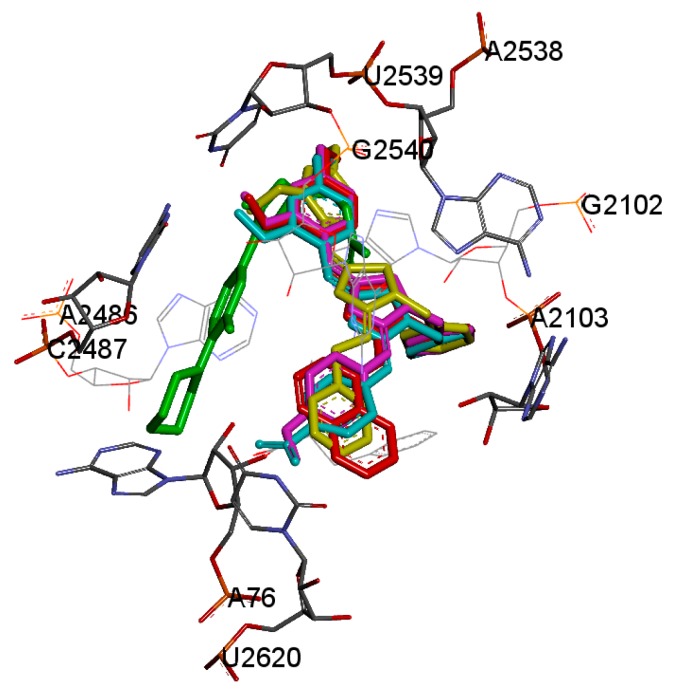
Binding mode of the **8f**–**i** series. Linezolid (green), **8f** (yellow), **8g** (red), **8h** (cyano), **8i** (magenta).

The results revealed that the binding mode of our compounds to the ribosome was similar to those of linezolid. The superposition of the docked configurations of the most active compounds, **8c** and **8h**, and *Haloarcula marismortui* LZD-bound 50S crystal structures is shown in Figure 2 and Figure 3.

In particular, the obtained data showed that our compounds bind the ribosomal unit between the P-site and A-site [34] in the same region of the Linezolid with binding free energies (ΔG_b_) in the range of −6.65 to −10.74 kcal/mol and in the same context hydrogen bond, π-π stacked and π-π T-shaped interactions were evaluated (see Supplementary Materials Table S1).

The docking data indicated that some compounds held deep into the active pocket by well established bonds as a combination of various hydrophobic and van der Waals interactions with one or more amino acids in the receptor active pocket of the enzyme. In particular, the results revealed two different binding modes, related to the nature of the substituent at the oxazolidine ring nitrogen atom. In particular, compounds **8a**–**e**, with **8c** showing the best binding free energy of the series, characterized by the presence of a methyl group at the oxazolidine nitrogen atom, located between the P-site (the binding site for peptidyl-tRNA) and the A-site (the binding site for incoming aminoacyl-tRNA), in the same way of linezolid (Figure 2), while the **8f**–**i** series, carrying a *N*-benzyl group at the nitrogen atom, appears to be shifted mainly to the A-site (Figure 3) [37,38].

**Figure 4:**
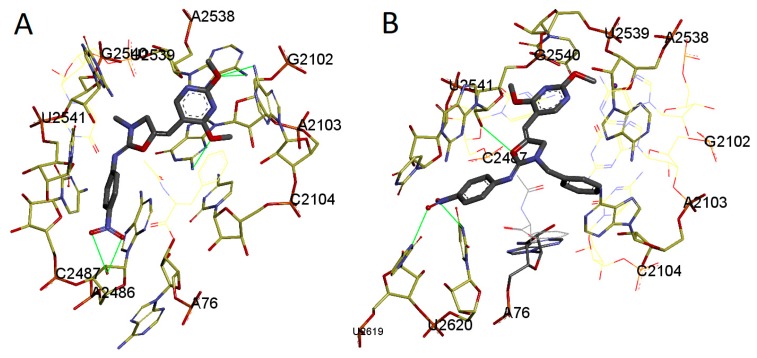
Molecular interaction of **8c** (**A**) and **8h** (**B**).

Among the synthesized compounds, pyrimidine-oxazolidinone hybrids **8c** (*N*-methyl series) and **8h** *(N*-benzyl series) exhibited high binding affinity (−9.65 and −10.74 kcal/mol, ΔG_b_), which was in agreement with their antibacterial activity. The docking pose of **8c** (Figure 4A) formed hydrogen bonds with the NH_2_ of A2538 and G2102 trough the oxygen atoms of the pyrimidine methoxy groups. A further hydrogen bond was found between the NO_2_ group and the 2′-sugar oxygen of A2486. The docking pose of **8h** (Figure 4B) formed hydrogen bonds between the oxazolidine ring oxygen atom and the 2′-sugar hydroxyl group of key residue G2540. In addition, hydrogen bonds were formed between the NO_2_ (aromatic ring) with NH (uracil) of the residues U2619 and U2620. Furthermore, π-π interactions were found between the pyrimidine and aromatic rings of the two compounds with the ribosomal residues.

**Figure 5:**
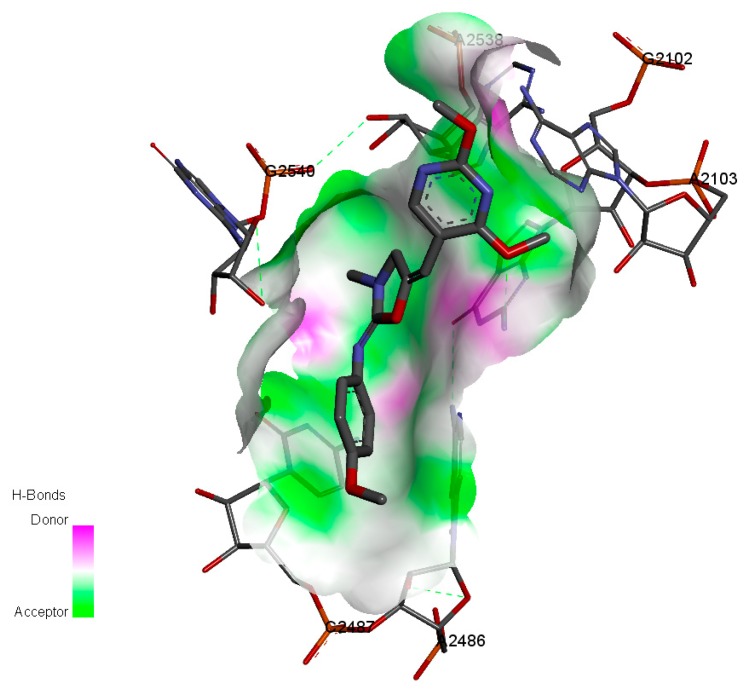
Docking pose of **8e**, H-bond receptor surface was displayed in green and magenta.

Conversely, the docking pose of **8e**, the less active compound, shows hydrogen bonds between the oxygen atoms of the pyrimidine methoxy groups and the NH_2_ of A2103 and G2102 and π-π interactions with the aromatic ring. However, the aromatic *p*-methoxy group is located in a hydrogen bond acceptor region, near the residues C2487 and A2486: this unfavourable interaction may account for the poor activity of this derivative (Figure 5).

## 3. Conclusions

The present research study reports the successful synthesis and antibacterial studies of a new class of pyrimidine-1,3-oxazolidin-2-arylimino hybrids carrying biologically active groups. The results of the biological screening revealed that all the compounds showed moderate to good activity against pathogenic strains. Among the screened compounds, derivatives **8c** and **8h** showed the most promising antibacterial activity: their good antimicrobial activity is accompanied with relatively low level of cytotoxicity, which reflects their therapeutic potential for their growth in the field of anti-infective agents.

Molecular docking study, performed in the crystal structure of 50S ribosomal unit of *Haloarcula marismortui,* showed that hydrogen bond and π–π interactions in the active site of the could explain the observed biological activity. It can be concluded that a combination of pyrimidine and oxazolidone moieties has led to new effective antimicrobial agents, thus suggesting that this scaffold can be considered as valid basis to develop of new further modifications to obtain more efficacious antibacterial compounds.

## 4. Materials and Methods

### 4.1. Chemistry

All chemicals were purchased from Sigma-Aldrich Chemical Co. The solvent was removed at aspirator pressure using a rotary evaporator. TLC was performed with Merck precoated TLC plates, and the compounds were made visible using a fluorescent inspection lamp and iodine vapor. Gravity chromatography was done with Merck silica gel 60 (mesh size 63–200 μm). Nuclear magnetic resonance spectra were recorded on a Varian Inova instrument, operating at 500 MHz for ^1^H NMR and 75 MHz for ^13^C NMR. Chemical shifts (δ) for ^1^H NMR spectra are reported in ppm downfield relative to the center line of CDCl_3_ triplet at 7.26 ppm. Chemical shifts for ^13^C NMR spectra are reported in ppm downfield relative to the center line of CDCl_3_ triplet at 77.23 ppm. The abbreviations s, d, t, and m stand for the resonance multiplicities singlet, doublet, triplet, and multiplet, respectively. ^13^C spectra, are ^1^H decoupled, and multiciplities were determined by APT pulse sequence. The melting points were recorded on a Boëtius hot plate microscope. FT-IR spectra were recorded on FT-IR Shimadzu spectrometer (4000–400 cm^−1^. EI-MS and HRMS were performed with Finnigan MAT 95, EI: 70 eV, R:10000.

### 4.2. General Procedure for the Preparation of (2Z,5E)-3-substituted-5-((2,4-dimethoxypyrimidin-5-yl)methylidene)-N-(aryl)oxazolidin-2-imino (***8a***–***i***)

K_3_PO_4_ (3M solution in H_2_O, 150 µL), the 2-4-dimethoxy-5-pyrimidinylboronic acid (2 mmol), and Pd(dppf)Cl_2_.CH_2_Cl_2_ (0.011 mmol) were added to a stirred solution of **7** (1 mmol) in THF (50 mL). The reaction mixture was refluxed at 60 °C overnight. The reaction mixture was concentrated in vacuo and the crude product was dissolved in CHCl_3_ (50 mL). The organic phase was washed with brine solution (3 × 30 mL), dried over MgSO_4_, filtered and the solvent removed under reduced pressure. The obtained product was purified by flash chromatography (AcOEt/*n*Hexane 20%) to yield final desired compounds **8a**–**i**.

*N*-{(5*E*)-5-[(2,4-dimethoxypyrimidin-5-yl)methylidene]-3-methyl-1,3-oxazolidin-2-ylidene} aniline (**8a**) Colorless oil (170 mg, 52%). Eluent: Cyclohexane/AcOEt 3/2. IR (neat) ν_max_/ cm^−1^: 1644 (C=N), 1581, 1384, 1275 (OMe). ^1^H NMR (500 MHz, CDCl_3_) δ 7.96 (s, 1H), 7.29–7.26 (m, 3H), 7.09–7.02 (m, 2H), 6.12 (t, 1H, *J* = 2.5 Hz), 4.32 (d, 2H, *J* = 2.5 Hz), 4.02 (s, 3H), 4.00 (s, 3H), 3.05 (s, 3H).^13^C NMR (125 MHz, CDCl_3_) δ 167.7 (s), 164.5 (s), 154.5 (d), 149.5 (s), 147.8 (s), 146.4 (s), 128.6 (d), 123.5 (d), 122.7 (d), 110.1 (s), 94.7 (d), 55.3 (q), 54.1 (q), 50.0 (t), 32.3 (q). HRMS-EI (*m*/*z*) [M^+^] calcd for C_17_H_18_N_4_O_3_ 326.1379 found 326.1375.

*N*-{(5*E*)-5-[(2,4-dimethoxypyrimidin-5-yl)methylidene]-3-methyl-1,3-oxazolidin-2-ylidene}-5,8-dihydronaphthalen-1-amine (**8b**) Colorless oil (263 mg, 70%). Eluent: Cyclohexane/AcOEt 3/2. IR (neat) ν_max_/ cm^−1^: 1641 (C=N), 1580, 1392, 1280 (OMe). ^1^H NMR (500 MHz, CDCl_3_): ^1^H NMR (500 MHz, CDCl_3_) δ 8.24–8.21 (m, 1H), 7.96 (s, 1H), 7.83–7.78 (m, 1H), 7.53 (d, *J* = 8.6 Hz, 1H), 7.47–7.38 (m, 3H), 7.17 (d, *J* = 7.8 Hz, 1H), 6.06 (t, *J* = 2.6, 1H), 4.38 (d, *J* = 2.6 Hz, 2H), 3.99 (s, 3H), 3.98 (s, 3H), 3.19 (s, 3H). ^13^C NMR (125 MHz, CDCl_3_) δ 166.8 (s), 162.3 (s), 154.8 (d), 152.6 (s), 146.9(s), 142.0 (s), 137.5 (s), 136.4 (s), 127.7 (d), 125.8 (d), 125.6 (d), 124.8 (d), 124.0 (d), 122.4 (s), 117.7 (d) 112.3 (s), 94.4 (d), 54.8 (q), 54.1 (q), 50.8 (t), 31.9 (q). HRMS-EI (*m*/*z*) [M^+^] calcd for C_21_H_20_N_4_O_3_ 376.1535 found 376.1538.

*N*-{(5*E*)-5-[(2,4-dimethoxypyrimidin-5-yl)methylidene]-3-methyl-1,3-oxazolidin-2-ylidene}-4-nitroaniline (**8c**) Colorless oil (286 mg, 77%). Eluent: Cyclohexane/AcOEt 3/2. IR (neat) ν_max_/ cm^−1^: 1641 (C=N), 1585, 1390, 1284(OMe). ^1^H NMR (500 MHz, CDCl_3_) δ 8.14 (d, *J* = 8.1 Hz, 2H), 7.97 (s, 1H), 7.16 (d, *J* = 8.1 Hz, 2H), 6.20 (t, *J* = 2.6 Hz, 1H), 4.39 (d, *J* = 2.6 Hz, 2H), 4.02 (s, 3H), 4.00 (s, 3H), 3.10 (s, 3H). ^13^C NMR (125 MHz, CDCl3) δ 167.8 (s), 164.2 (s), 155.1 (d), 152.5 (s), 150.7(s), 149.9 (s), 146.6 (s), 127.7 (d), 124.6 (d), 109.1 (s), 95.6 (d), 54.9 (q), 54.1 (q), 50.5 (t), 31.7 (q). HRMS-EI (*m*/*z*) [M^+^] calcd for C_17_H_17_N_5_O_5_ 371.1230 found 371.1228.

3-chloro-*N*-{(5*E*)-5-[(2,4-dimethoxypyrimidin-5-yl)methylidene]-3-methyl-1,3-oxazolidin-2-ylidene}aniline (**8d**) Colorless oil (259 mg, 72%). Eluent: Cyclohexane/AcOEt 3/2. IR (neat) ν_max_/ cm^−1^: 1641 (C=N), 1582, 1390, 1282 (OMe). ^1^H NMR (500 MHz, CDCl_3_) δ 7.96 (s, 1H), 7.21(d, *J* = 7.8 Hz, 1H), 7.11 (d, *J* = 6.8 Hz, 1H), 6.96–6.93 (m, 2H), 6.16 (t, *J* = 2.6 Hz, 1H), 4.34 (d, *J* = 2.6 Hz, 2H), 4.01 (s, 3H), 3.99 (s, 3H), 3.10 (s, 3H). ^13^C NMR (125 MHz, CDCl_3_) δ 167.5 (s), 164.6 (s), 157.8 (d), 155.2 (s), 151.7(s), 146.7 (s), 133.9 (s), 130.8 (d), 124.3 (d), 122.8 (d), 119.8 (d), 114.7 (s), 96.1 (d), 55.4 (q), 54.2 (q), 50.0 (t), 34.2 (q). HRMS-EI (*m*/*z*) [M^+^] calcd for C_17_H_17_ClN_4_O_3_ 360.0989 found 360.0991.

*N*-{(5*E*)-5-[(2,4-dimethoxypyrimidin-5-yl)methylidene]-3-methyl-1,3-oxazolidin-2-ylidene}-4-methoxyaniline (**8e**) Colorless oil (157 mg, 44%). Eluent: Cyclohexane/AcOEt 3/2. IR (neat) ν_max_/ cm^−1^: 1641 (C=N), 1580, 1380, 1270 (OMe). ^1^H NMR (500 MHz, CDCl_3_) δ 7.96 (s, 1H), 7.34 (d, *J* = 8.0 Hz, 2H), 6.84 (d, *J* = 8.0 Hz, 2H), 6.23 (t, *J* = 2.4 Hz, 1H), 4.43 (d, *J* = 2.4 Hz, 2H), 3.95 (s, 3H), 3.87 (s, 3H), 3.78 (s, 3H) 3.07 (s, 3H).^13^C NMR (125 MHz, CDCl_3_) δ^13^C NMR (125 MHz, CDCl_3_) δ 166.2 (s), 163.5 (s), 156.4 (d), 155.9 (s), 153.2 (s), 146.9 (s), 146.2 (s), 124.1 (d), 116.4 (d), 114.9 (s), 95.7 (d), 55.2 (q), 55.4 (q), 54.3 (q), 49.9 (t), 34.3 (q). HRMS-EI (*m*/*z*) [M^+^] calcd for C_18_H_20_N_4_O_4_ 356.1485 found 356.1483.

*N*-[(5*E*)-5-{[4-(benzyloxy)-2-methoxypyrimidin-5-yl]methylidene}-3-methyl-1,3-oxazolidin-2-ylidene]-1-phenylmethanamine (**8f**) Colorless oil (187 mg, 45%). Eluent: Cyclohexane/AcOEt 3/2. IR (neat) ν_max_/ cm^−1^: 1641 (C=N), 1580, 1380, 1270 (OMe). ^1^H NMR (500 MHz, CDCl_3_) δ 7.86 (s, 1H), 7.41–7.39 (m, 2H), 7.37–7.28 (m, 7H), 7.24–7.21 (m, 1H), 6.13 (t, *J* = 2.4 Hz, 1H), 4.59 (s, 2H), 4.55 (s, 2H), 4.08 (d, *J* = 2.4 Hz, 2H), 3.98 (s, 3H), 3.96 (s, 3H). ^13^C NMR (125 MHz, CDCl_3_) δ 166.2 (s), 163.5 (s), 157.8 (d), 155.2 (s), 146.4 (s), 142.3 (s), 137.5 (s), 129.7 (s), 129.2 (d), 128.2 (d), 127.3 (d), 107.9 (d), 95.4 (d), 54.8 (q), 54.1 (q), 51.6 (t), 51.3 (t), 46.7 (t). HRMS-EI (*m*/*z*) [M^+^] calcd for C_24_H_24_N_4_O_3_ 416.1848 found 416.1843.

*N*-{(5*E*)-3-benzyl-5-[(2,4-dimethoxypyrimidin-5-yl)methylidene]-1,3-oxazolidin-2-ylidene}-5,8-dihydronaphthalen-1-amine (**8g**) Colorless oil (334 mg, 74%). Eluent: Cyclohexane/AcOEt 3/2. IR (neat) ν_max_/ cm^−1^: 1641 (C=N), 1578, 1391, 1281 (OMe). ^1^H NMR (500 MHz, CDCl3) δ 7.76 (d, *J* = 6.6 Hz, 1H), 7.37 (s, 1H), 7.32 (d, *J* = 6.2, Hz, 1H), 7.06 (d, *J* = 8.2 Hz, 1H), 6.99–6.83 (m, 7H), 6.75 (d, *J* = 0.7 Hz, 2H), 5.58 (t, 1H), 4.30 (s, 2H), 3.74 (d, *J* = 2.5 Hz, 2H), 3.46 (s, 3H), 3.45 (s, 3H). ^13^C NMR (125 MHz, CDCl_3_) δ 165.9 (s), 163.3 (s), 154.6 (d),149.2 (s), 149.9 (s), 142.9 (s), 135.8 (s), 134.9 (s), 129.4 (d), 128.9 (d), 128.3 (d), 128.0 (d), 127.7 (d), 127.5 (d), 126.5 (s), 125.9 (d), 125.6 (d), 124.9 (d), 124.1 (d), 117.7 (s), 94.6 (d), 54.8 (q), 54.1 (q), 48.9 (t), 47.9 (t). HRMS-EI (*m*/*z*) [M^+^] calcd for C_27_H_24_N_4_O_3_ 452.1848 found 452.1845.

*N*-{(5*E*)-3-benzyl-5-[(2,4-dimethoxypyrimidin-5-yl)methylidene]-1,3-oxazolidin-2-ylidene}-4-nitroaniline (**8h**) Colorless oil (389 mg, 87%). Eluent: Cyclohexane/AcOEt 3/2. IR (neat) ν_max_/ cm^−1^: 1641 (C=N), 1588, 1390, 1240 (OMe). ^1^H NMR (500 MHz, CDCl3) δ 8.20–8.16 (t, *J* = 8.1 Hz, 2H), 7.88 (s, 1H), 7.41–7.33 (m, 5H), 7.24–7.20 (m, 1H), 6.20 (t, 1H), 4.67 (s, 2H), 4.22 (d, *J* = 2.5 Hz, 2H), 3.98 (s, 3H), 3.97 (s, 3H).^13^C NMR (125 MHz, CDCl_3_) δ 168.6 (s), 164.5 (s), 158.6 (d), 153.5 (s), 150.4 (s), 146.6 (s), 142.5 (s), 135.0, (s), 128.9 (d), 128.2 (d), 124.6 (d), 123.8 (d), 108.5 (s), 95.6 (d), 54.8 (q), 54.1 (q), 48.6 (t), 47.6 (t). HRMS-EI (*m*/*z*) [M^+^] calcd for C_23_H_21_N_5_O_5_ 447.1543 found 447.1541.

*N*-{(5*E*)-3-benzyl-5-[(2,4-dimethoxypyrimidin-5-yl)methylidene]-1,3-oxazolidin-2-ylidene}-4-methoxyaniline (**8i**) Colorless oil (216 mg, 50%). Eluent: Cyclohexane/AcOEt 3/2. IR (neat) ν_max_/ cm^−1^: 1641 (C=N), 1598, 1558, 1464, 1238 (OMe). ^1^H NMR (500 MHz, CDCl_3_) δ 7.86 (s, 1H), 7.36–7.31 (m, 4H), 7.09 (d, *J* = 9.5 Hz, 2H), 6.85 (d, *J* = 9.5 Hz, 2H), 6.13 (t, *J* = 2.9 Hz, 1H), 4.63 (s, 2H), 4.12 (d, *J* = 2.9 Hz, 2H), 3.97 (s, 3H), 3.96 (s, 3H), 3.84 (s, 3H). ^13^C NMR (125 MHz, CDCl_3_) δ 166.5 (s), 164.3 (s), 157.8 (d), 155.9 (s), 150.2 (s), 146.5 (s), 145.2 (s), 137.4, (s), 129.3 (d), 129.0 (d), 128.9 (d), 123.8 (d), 116.4 (d), 114.9 (s), 95.5 (d), 56.2 (q), 55.4 (q), 50.1 (q), 51.6 (t), 46.7 (t). HRMS-EI (*m*/*z*) [M^+^] calcd for C_24_H_24_N_4_O_4_ 432.1798 found 432.1795.

## Figures and Tables

**Table 1:** Alkoxyiodination of alkynyl ureas.

Entry	Urea	R	Ar	Product (Yield %)
1	**6a**	Me	Ph	**7a** (63%)^23^
2	**6b**	Me	1-naphthyl	**7b** (73%)^23^
3	**6c**	Me	4-NO_2_-C_6_H_4_	**7c** (68%)^23^
4	**6d**	Me	3-Cl-C_6_H_4_	**7d** (75%)
5	**6e**	Me	4-MeO-C_6_H_4_	**7e** (60%)
6	**6f**	Bn	Bn	**7f** (63%)
7	**6g**	Bn	1-naphthyl	**7g** (79%)
8	**6h**	Bn	4-NO_2_-C_6_H_4_	**7h** (76%)
9	**6i**	Bn	4-MeO-C_6_H_4_	**7i** (42%)

**Table 2:** Suzuki reaction of 5-(Iodomethylydene)-2-imino-oxazolidines **7a**–**i** with 2,4-dimethoxypirimidin-5-boronic acid.

Entry	Oxazolidines	R	Ar	Product (Yield %)
1	**7a**	Me	Ph	**8a** (52%)
2	**7b**	Me	1-naphthyl	**8b** (70%)
3	**7c**	Me	4-NO_2_-C_6_H_4_	**8c** (77%)
4	**7d**	Me	3-Cl-C_6_H_4_	**8d** (72%)
5	**7e**	Me	4-MeO-C_6_H_4_	**8e** (44%)
6	**7f**	Bn	Bn	**8f** (45%)
7	**7g**	Bn	1-naphthyl	**8g** (74%)
8	**7h**	Bn	4-NO_2_-C_6_H_4_	**8h** (87%)
9	**7i**	Bn	4-MeO-C_6_H_4_	**8i** (50%)

**Table 3:** Minimum inhibitory concentration (MIC) (μg/mL) for compounds **8a**–**i**.

Compounds	Gram-Positive Bacteria		Gram-Negative Bacteria			Fungi	
	Bs	Sa	Pa	St	Kb	Ca	Ct
**8a** (R=Me; Ar=Ph)	36	45	48	40	120	250	800
**8b** (R=Me; Ar=1-Naft)	14	13	24	13	48	140	140
**8c** (R=Me; Ar=p-NO_2_)	4.2	4.8	13	21	13	24	100
**8d** (R=Me; Ar=3-Cl)	300	600	800	400	80	1000	1000
**8e** (R=Me; Ar=p-OMe)	400	800	800	1000	600	800	1000
**8f** (R=Bn; Ar=Bn)	9.5	7.5	24	24	14	140	140
**8g** (R=Bn; Ar=1-Naft)	10	12	48	48	24	110	140
**8h** (R=Bn; Ar=p-NO_2_)	3.2	2.8	9.5	12	12	14	100
**8i** (R=Bn; Ar=p-OMe)	24	19	48	28	30	85	105
Linezolid	3	4	3	4	-	-	-
Ciprofloxacin	3.5	3.5	3	3	-	-	-
Fluconazole	-	-	-	-	-	20	12

**Table 4:** Cytotoxicity levels of selected compounds on HDF cell line.

Compound	CC_50_ (μg/mL)
**8b**	98
**8c**	>200
**8f**	120
**8g**	120
**8h**	>200

## Supplementary Materials

The supplementary materials are available online. Supporting information includes experimental procedures and characterization data of the compounds described in this article. Table S1: Binding free energy (ΔG_b_) and molecular interactions of compounds **8a**–**i**.
