# Supplementary material for: Synthesis and Biological Evaluation of Pyrimidine-oxazolidin-2-arylimino Hybrid Molecules as Antibacterial Agents
Source: Molecules. 2018 Jul 17;23(7):1754. doi: 10.3390/molecules23071754 (PMC6099984; doi:10.3390/molecules23071754)
Supplement: Supplementary file 1 [file molecules-23-01754-s001.pdf]

Supporting Information

Synthesis and Biological Evaluation of Pyrimidine-Oxazolidin-2-arylimino hybrid molecules as Antibacterial Agents

Roberto Romeo,[a*] Maria A. Chiacchio,[b] Agata Campisi,[b] Giulia Monciino, Lucia Veltri,[c] Daniela Iannazzo,[d] Gianluigi Broggini[e] and Salvatore V. Giofrè[a]

[a] Dipartimento di Scienze chimiche, biologiche, farmaceutiche ed ambientali, Università di Messina, Via S.S. Annunziata, 98168 Messina, Italy. **Email:** robromeo@unime.it

[b] Dipartimento di Scienze del farmaco, Università di Catania, Viale A. Doria, 95100 Catania, Italy.

[c] Dipartimento di Chimica e tecnologie chimiche, Università della Calabria, Via P. Bucci, 12/C, 87036 Arcavacata di Rende, Italy.

[d] Dipartimento di Ingegneria, Università di Messina, Contrada Di Dio, 98166 Messina, Italy.

[e] Dipartimento di Scienza e Alta Tecnologia, Università dell'Insubria, via Valleggio 11, 22100 Como, Italy

Table of Contents

General S3

General procedure for syntheses of **7a-i**...S3

Biological assay...S4

Molecular Docking Study S5

General

All chemicals were purchased from Sigma–Aldrich Chemical Co. The solvent was removed at aspirator pressure using a rotary evaporator. TLC was performed with Merck precoated TLC plates, and the compounds were made visible using a fluorescent inspection lamp and iodine vapor. Gravity chromatography was done with Merck silica gel 60 (mesh size 63–200 ◎m). Nuclear magnetic resonance spectra were recorded on a Varian Inova instrument, operating at 500 MHz for ^{1}H NMR and 75 MHz for ^{13}C NMR. Chemical shifts (◎) for ^{1}H NMR spectra are reported in ppm downfield relative to the center line of $CDCl_3$ triplet at 7.26 ppm. Chemical shifts for ^{13}C NMR spectra are reported in ppm downfield relative to the center line of $CDCl_3$ triplet at 77.23 ppm. The abbreviations s, d, t, and m stand for the resonance multiplicities singlet, doublet, triplet, and multiplet, respectively. ^{13}C spectra, are ^{1}H decoupled, and multiciplities were determined by APT pulse sequence. The melting points were recorded on a Boëtius hot plate microscope. FT-IR spectra were recorded on FT-IR Shimadzu spectrometer (4000–400 cm^{-}1. EI-MS and HRMS were performed with Finnigan MAT 95, EI: 70 eV, R:10000.

General procedure for syntheses of 5-[(iodomethylidene)-2-arylimino]oxazolidines (7a-i).

A solution of alkynyl amide (**6**) (1 mmol), CuI_2 (0.05 mmol) and NIS (1 mmol) in MeCN (15 ml) was stirred under nitrogen at 70 °C for 7 h and then left at room temperature overnight. The reaction mixture turns red-brown and a precipitate appears. After the completion of the reaction, the mixture was filtered on a pad of silica gel, and washed with EtOAc until the silica appeared uncolored. The solvent was removed under reduced pressure and the crude product was purified by silica gel column chromatography to yield the final desired compounds **7a**-**i**.

(E)-5(iodomethylidene)-3-methyl-2-(phenylimino)oxazolidine (**7a**).
This compound was found to be identical to authentic samples already reported in literature, synthesized using $CuCl_2$ as catalytic system.[23]

(E)-5(iodomethylidene)-2-[(1-naphthyl)imino]-3-methyloxazolidine (**7b**).
This compound was found to be identical to authentic samples already reported in literature, synthesized using $CuCl_2$ as catalytic system.[23]

(E)-5(iodomethylidene)-3-methyl-2-[(4-nitrophenyl)imino]oxazolidine(**7c**).
This compound was found to be identical to authentic samples already reported in literature, synthesized using $CuCl_2$ as catalytic system.[23]

(E)- 2-[(3-Chlorophenyl)imino]-5-(Iodomethylidene)-3-methyloxazolidine(**7d**).
Pale yellow oil (261 mg, 75%). Eluent: Cyclohexane/AcOEt 3/2. ◎$_{max}$/ cm^{-1}: 1647 (C=N). ^{1}H NMR (500 MHz, $CDCl_3$) δ 7.15 (t, *J* = 7.0 Hz, 1H), 7.04 (d, *J* = 1.7 Hz, 1H), 6.96 (d, J = 7.9 Hz, 1H), 6.90 (d, J = 7.9 Hz, 1H), 5.68 (t, *J* = 1.4 Hz, 1H), 4.08 (d, *J* = 1.4 Hz, 2H), 2.98 (s, 3H). ^{13}C NMR (125 MHz, $CDCl_3$) δ 150.2 (s), 150.1 (s), 147.4 (s), 133.6 (s), 129.3 (d), 123.4 (d), 122.3

(d), 121.7 (d), 53.0 (t), 49.6 (q), 31.3 (q). HRMS-EI (m/z) [M^+] calcd for $C_{11}H_{10}ClIN_2O$ 347.9526 found 347.9523.

(E)-5(iodomethylidene)-2-[(4-methoxyphenyl)imino]-3-methyloxazolidine (**7e**).
Colorlessoil (206 mg, 60%). Eluent: Cyclohexane/AcOEt 7/2. ◎$_{max}$/ cm^{-1}: 1654 (C=N). ^{1}H NMR (500 MHz, $CDCl_3$): δ 6.97 (d, *J* = 8.5 Hz, 2H), 6.80 (d, *J* = 8.5 Hz, 2H), 5.63 (t, *J* = 2.5 Hz, 1H), 4.09 (d, J = 2.5 Hz, 2H), 3.76 (s, 3H), 2.98 (s, 3H). ^{13}C NMR (125 MHz, $CDCl_3$) δ 155.1 (s), 150.7 (s), 149.7 (s), 139.2 (s), 124.2 (d), 113.7 (d), 55.3 (q), 53.3 (t), 48.8 (d), 31.6 (q). HRMS-EI (m/z) [M^+] calcd for $C_{12}H_{13}INO_2$ 344.0022 found 344.0025.

(E)-3-benzyl-2-[(benzyl)imino]-5-(iodomethylidene)oxazolidine (**7f**).
Colorlessoil (254 mg, 63%). Eluent: Cyclohexane/AcOEt 5/2. ◎$_{max}$/ cm^{-1}: 1652 (C=N). ^{1}H NMR (500 MHz, $CDCl_3$): δ 7.37 – 7.29 (m, 8H), 7.23 –7.18 (m, 2H), 5.62 (t, *J* = 2.6 Hz, 1H), 4.52 (d, *J* = 2.6 Hz, 2H), 3.81 (s, 2H), 3.79 (s, 2H). ^{13}C NMR (125 MHz, $CDCl_3$) δ 155.4 (s), 154.5 (s), 142.0 (s), 137.4 (s), 129.5 (d), 129.2 (d), 129.0 (d), 128.3 (d), 127.7 (d), 52.3 (d), 51.5 (t), 51.4 (t), 49.5 (t). HRMS-EI (m/z) [M^+] calcd for $C_{18}H_{17}IN_2O$ 404.0386 found 404.0388

(E)-3-benzyl-5(iodomethylidene)-2-[(1-naphtyl)imino]oxazolidine(**7g**).
Colorlessoil (348 mg, 79%). Eluent: Cyclohexane/AcOEt 3/1. ◎$_{max}$/ cm^{-1}: 1656 (C=N). ^{1}H NMR (500 MHz, $CDCl_3$): δ 7.90 (d, *J* = 8.4 Hz, 1H), 7.52 (d, J = 8.5 Hz, 1H), 7.44 –7.32 (m, 8H), 7.16 (d, 1H), 6.91 (d, *J* = 8.4 Hz, 1H), 5.56 (t, J = 2.6 Hz, 1H), 4.72 (s, 2H), 4.00 (t, J = 2.6 Hz, 2H).^{13}C NMR (125 MHz, $CDCl_3$) δ 156.5 (s), 154.3 (s), 147.2 (s), 137.4 (s), 136.5 (s), 129.3 (d), 129.1 (d), 128.9 (d), 127.9, (d), 127.5 (d), 127.0 (d), 126.5 (s), 125.8 (d), 124.2 (d), 122.4 (d), 121.2 (d), 52.4 (d), 51.5 (t), 49.6 (t). RMS-EI (m/z) [M^+] calcd for $C_{21}H_{17}IN_2O$ 440.0386 found 440.0389.

(E)-3-Benzyl-5(iodomethylidene)-2-[(4-nitrophenyl)imino]oxazolidine (**7h**).
Colorlessoil (330 mg, 76%). Eluent: Cyclohexane/AcOEt 3/2. ◎$_{max}$/ cm^{-1}: 1641 (C=N). ^{1}H NMR (500 MHz, $CDCl_3$): δ 8.15 (d, *J* = 9.0 Hz, 2H), 7.43 – 7.33 (m, 5H), 7.16 (d, J = 9.0 Hz, 2H), 5.77 (t, *J* = 2.7 Hz, 1H), 4.64 (s, 2H), 4.04 (t, *J* = 2.7 Hz, 2H). ^{13}C NMR (125 MHz, $CDCl_3$) δ 156.4 (s), 154.9 (s), 154.4 (s), 141.6 (s), 137.3 (s), 129.3 (d), 129.0 (d), 128.9 (d), 126.2, (d), 123.5 (d), 52.7 (d), 51.8 (t), 49.4 (t). HRMS-EI (m/z) [M^+] calcd for $C_{17}H_{14}IN_3O_3$ 435.0080 found 435.0081.

(E)-3-Benzyl-5(iodomethylidene)-2-[(4-methoxyphenyl)imino]oxazolidine (**7i**).
Pale yellow oil (176 mg, 42%). Eluent: Cyclohexane/AcOEt 3/2. ◎$_{max}$/ cm^{-1}: 1644 (C=N). ^{1}H NMR (500 MHz, $CDCl_3$): δ 7.66 (t, *J* = 6.2 Hz, 1H), 7.52 (t, *J* = 7.8 Hz, 2H), 7.41 – 7.31 (m, 2H), 7.02 (d, *J* = 9 Hz, 2H), 6.82 (d, *J* = 9 Hz, 2H), 5.65 (t, J = 2.6 Hz, 1H), 4.62 (s, 2H), 3.97 (d, *J* = 2.6 Hz, 2H), 3.79 (s, 3H). ^{13}C NMR (125 MHz, $CDCl_3$) δ 155.9 (s), 154.6 (s), 153.8 (s), 145.3 (s), 137.4 (s), 129.2 (d), 128.9 (d), 128.0 (d), 123.9, (d), 116.5 (d), 56.2 (q), 52.4 (d), 51.4 (t), 49.6 (t). HRMS-EI (m/z) [M^+] calcd for $C_{18}H_{17}IN_2O_2$ 420.0335 found 420.0337.

Biological assay

Antimicrobial al activity. MIC testing

The MIC values were tested using the agar dilution method according to the National Committee for Clinical Laboratory Standards (NCCLS, 4th ed).[24] Briefly, compounds were dissolved in 25% DMSO to a solution with concentration of 1280 μg/mL. Ciprofloxacin and fluconazol, used as reference drugs, were directly dissolved in 10 mL distilled water. All the solutions were then diluted two-fold with distilled water to provide 11 dilutions (final concentration is 0.625 μg/mL). A 2 mL volume of the 2-fold serial dilution of each test compound/drug was incorporated into 18 mL hot MuellereHinton agar medium, which resulted in the final concentration of each dilutions decreasing ten fold. The studied microorganisms were maintained at 4°C. The Gram positive bacteria studied were *Bacillus subtilis* MRCC121 and *Staphylococcus aureus* MTCC96. Gram negative bacteria were *Pseudomonas aeruginosa* MTCC741, *Salmonella typhi* MTT537, *Klebsiella pneumoniae* MTCC3384 and fungal strains were *Candida albicans* MTCC3017 and *Candida tropicalis* MTCC184. A 10 μL amount of bacterial suspension was spotted onto Muellere-Hinton agar plates containing serial dilutions of the compounds/drug. The plates were incubated at 36.5 °C for 24–48 h. The same procedure was repeated in triplicate.

In vitro toxicity study

Cells and Reagents

Human Dermal Fibroblasts (HDF), Basal Medium and Fibroblast Growth Supplement (FGS) were obtained from Innoprot (Derio–Bizkaia, Spain). Poly-L-lysine, 3(4,5-dimethyl-thiazol-2-yl)2,5-diphenyl-tetrazolium bromide salts (MTT), dimethyl sulfoxide (DMSO) and other chemicals of analytical grade were obtained from Sigma–Aldrich (Milano, Italy). Heat inactivated- Foetal Bovine Serum (FBS, GIBCO), L-Glutamine, Phosphate Buffer Saline (PBS), Streptomycin and penicillin antibiotics, Trypsin–EDTA 0.05% solution were purchased from Thermo Fisher Scientific (USA).

MTT Bioassay

To monitor cell viability, HDF cell line was set up 60×10^4 cells/well of a 96 multiwell flat bottomed 200 μl microplates pre-coated with 1% Poly-L-lysine. Cells were incubated at 37°C in a humidified 5% CO_2–95 % air mixture. A lot of cell line was treated with different concentrations (1-100 μM) of **8c** or **8h** for 24h. At the end of treatment time, 20 μl MTT 0.5 %, in PBS was added to each multiwell. After 1 h of incubation with the reagent, the supernatant was removed and replaced with 100 μl DMSO. The optical density of each well sample was measured with a microplate spectrophotometer reader (TitertekMultiskan; Flow Laboratories, Helsinki, Finland) at λ 570 nm. The concentration of compound required to cause 50 % inhibition of cell proliferation (IC_{50}) was calculated from concentration–effect curves using Prism 5.0 (GrafPAD Software for Science). For all compounds, a non-linear regression analysis was used.

Molecular Docking Study

Receptor and Ligands Preparation

The X-ray crystal structure of 50S ribosomal unit of *Haloarculamarismortui*(PDB code 3CPW)enzyme conjugated with linezolid (PDB code 3CPW), with resolution of 2.7 Å, was retrieved from the protein data bank database (http://www.rcsb.org/pdb). The Accelrys Discovery Studio software has been used to decresing complexity of protein structure.All the atoms within30 Å distance from bound linezolid were selected and the relevantresidues were expanded. The selected construct was employed to molecular docking.The AutoDock Tools software has been used to remove ligands and water molecules, adding all the hydrogen atoms, and to calculate Gaisteiger charges for each atom of the macromolecules. The chemical structures of compounds were generated using ChemOffice v12.0 Ultra software package and optimized with ADT 4.2 for docking studies.

Docking Protocol

Molecular Docking was carried out using AutoDock 4.2. Grid Maps were centered on the coordinates of the co-cristallized ligands with 50 points per dimension and a step size of 0.375. The Lamarckian genetic algorithm implemented in AutoDock has been employed to dock compounds **8a-i** into the50S ribosomal unit. We have carried out comparative docking experiments of compounds **8a-i**. Each docking experiment consisted of 100 docking runs with 150 individuals and 2.5 x 10^5 energy evaluations. Other parameters were left to their default values. The search was conducted in a grid of 50 points per dimension and a step size of 0.375 centered on the binding site of enzyme. 100 highest-scoring docking poses were saved and binding affinity of the best mode was selected.The cluster with the lowest free energy of binding was visually analyzed using Python Molecular Viewer 1.4.3 (PMV).The Autodock docking parameters were validated to ensure that the ligand orientation and the position obtained from the docking studies represent valid reasonable binding modes of inhibitors. The ligand linezolid was extracted and docked back into the corresponding binding pocket. The results of docking simulation predicted the binding conformation of linezolid with a root mean square deviation (RMSD) of 0.76 Å, compared with conformation of co-crystallized structure, thus indicating a valid docking protocol.

Table S1. Binding free energy (◎G_b) and molecular interactions of compounds **8a-i**

Compound	⚜G_b (kcal/mol)	Interaction	Atom of Compound	Nucleotide	Distance(Å)
**8a**	-7.07	H-bonding	N (pyrimidine ring)	A2538	2.89
		H-bonding	O (4-OMe pyrimidine)	A2538	3.07
		Pi-Pi stacked	Aromatic ring	C2487	
**8b**	-8.18	H-bonding	N (pyrimidine ring)	A2538	2.93
		H-bonding	O (4-OMe pyrimidine)	A2538	3.00
		Pi-Pi stacked	Aromatic ring	C2487	
**8c**	-9.65	H-bonding	O (4-OMe pyrimidine)	A2538	3.33
		H-bonding	O (NO2)	A2486	2.93
		H-bonding	O (2-OMe pyrimidine)	G2102	2.91
		H-bonding	O (4-OMe pyrimidine)	A2103	2.85
		Pi-Pi T-shaped	Pyrimidine ring	A2103	
		Pi-Pi stacked	Aromatic ring	A2486	
**8d**	-7.41	H-bonding	O (4-OMe pyrimidine)	A2538	3.10
		H-bonding	N (pyrimidine ring)	A2538	2.92
		Pi-Pi stacked	Aromatic ring	C2487	
**8e**	-6.65	H-bonding	O (2-OMe pyrimidine)	G2102	3.03
		H-bonding	O (4-OMe pyrimidine)	A2103	2.71
		Pi-Pi T-shaped	Pyrimidine ring	A2103	
		Pi-Pi stacked	Aromatic ring	C2487	
		Pi-Pi stacked	Aromatic ring	A2486	
**8f**	-8.24	H-bonding	O (oxazolidine)	G2540	2.81
		Pi-Pi stacked	Pyrimidine ring	G2102	
		Pi-Pi T-shaped	Pyrimidine ring	U2539	
		Pi-Pi T-shaped	Aromatic ring (N-Benzyl)	A2539	
		Pi-Pi T-shaped	Aromatic ring (N-Benzyl)	A2103	
		Pi-Pi T-shaped	Aromatic ring	U2541	
**8g**	-8.96	H-bonding	O (oxazolidine)	G2540	3.02

		Pi-Pi T-shaped	Naphthyl ring	U2541	
		Pi-Pi T-shaped	Pyrimidine ring	U2539	
		Pi-Pi stacked	Pyrimidine ring	G2102	
		Pi-Pi T-shaped	Aromatic ring	A2538	
**8h**	-10.74	H-bonding	O (oxazolidine)	G2540	3.17
		H-bonding	O (NO_2)	U2619	3.10
		H-bonding	O (NO_2)	U2620	2.66
		Pi-Pi T-shaped	Pyrimidine ring	G2539	
		Pi-Pi stacked	Pyrimidine ring	G2102	
		Pi-Pi T-shaped	Aromatic ring (Benzyl)	A2538	
**8i**	-8.82	H-bonding	O (oxazolidine)	G2540	3.10
		Pi-Pi T-shaped	Pyrimidine ring	U2539	
		Pi-Pi stacked	Pyrimidine ring	G2102	
		Pi-Pi T-shaped	Aromatic ring (Benzyl)	A2538	
		Pi-Pi T-shaped	Aromatic ring (Benzyl)	A2103	
**Linezolid**	-7.87	H-bonding	N (acetamide)	G2540	2.85, 3.38
		H-bonding	F	G2540	3.21
		Pi-Pi stacked	Aromatic ring	C2487	
